# Case report: meningeal lymphangiogenesis around ependymoma forming along the dura matter

**DOI:** 10.3389/fonc.2023.1340167

**Published:** 2024-01-12

**Authors:** Utaro Hino, Ryota Tamura, Masahiro Yo, Yoshitaka Kase, Noboru Tsuda, Tsubasa Miyauchi, Junki Sogano, Kosuke Karatsu, Tomoru Miwa, Masahiro Toda

**Affiliations:** ^1^ Department of Neurosurgery, Keio University School of Medicine, Shinjuku-ku, Japan; ^2^ Department of Physiology, Keio University School of Medicine, Shinjuku-ku, Japan; ^3^ Department of Clinical Regenerative Medicine, School of Medicine, Fujita Health University, Toyoake-shi, Japan; ^4^ Department of Pathology, Keio University School of Medicine, Shinjuku-ku, Japan

**Keywords:** case report, ependymoma, dura, lymphangiogenesis, podoplanin

## Abstract

Recently, there has been growing interest in the presence and function of meningeal lymphatic vessels, with no direct evidence linking these vessels to primary brain tumors. We report a unique case of recurrent ependymoma in the dura mater, showing histopathological signs of lymphatic proliferation at the tumor attachment site. The patient initially presented with a headache, and was diagnosed with *ZFTA* fusion-positive supratentorial ependymoma, central nervous system WHO Grade 3. Following multiple dura mater recurrences and surgery, the fifth procedure revealed numerous tumors contralateral to the original site, with genetic testing confirming *ZFTA* fusion positivity, indicating recurrent ependymoma. Immunohistochemical analysis showed D2-40^+^ lymphatic vessel proliferation around tumor attachment sites within the dura mater. Elevated expression of *ZEB1*, which is an epithelial-to-mesenchymal transition factor, was also observed, implicating potential involvement in the unique pathophysiology. The present case suggests a new process of metastasis through meningeal lymphatic vessels, although we were unable to visually confirm tumor cell infiltration into the lymphatic vessels. This case is the first report suggesting ependymoma metastasis through dural lymphatic vessels, underlining the need for further case accumulation and study to understand the mechanisms of this phenomenon.

## 1 Introduction

Since the first discovery of lymphatic vessels in the dura mater in 1787, basic research on this structure continues to be reported (1). In recent decades, two independent studies have demonstrated the presence of functioning lymphatic vasculature in the extensive dura mater, draining cerebrospinal fluid (CSF), macromolecules, and immune cells from the meningeal spaces (2) and the brain parenchyma (3). Lymphatic vessels are located on each side of the dural sinuses, such as the superior sagittal sinus and transverse sinus, suggesting a new pathway for the movement of interstitial fluid from the brain parenchyma to the cervical lymph nodes. However, the function of the dural lymphatic vessels is not fully understood. Several reports have shown its association with neurodegenerative diseases such as Alzheimer’s disease (4) and multiple sclerosis (5). In this report, we describe an ependymoma that recurred repeatedly in the dura mater and histopathologically showed lymphangiogenesis at the site of tumor attachment. Tumor invasion through lymphatic vessels may need to be considered in the future as a novel route of progression of brain tumors.

## 2 Case description

**Figure 1:**
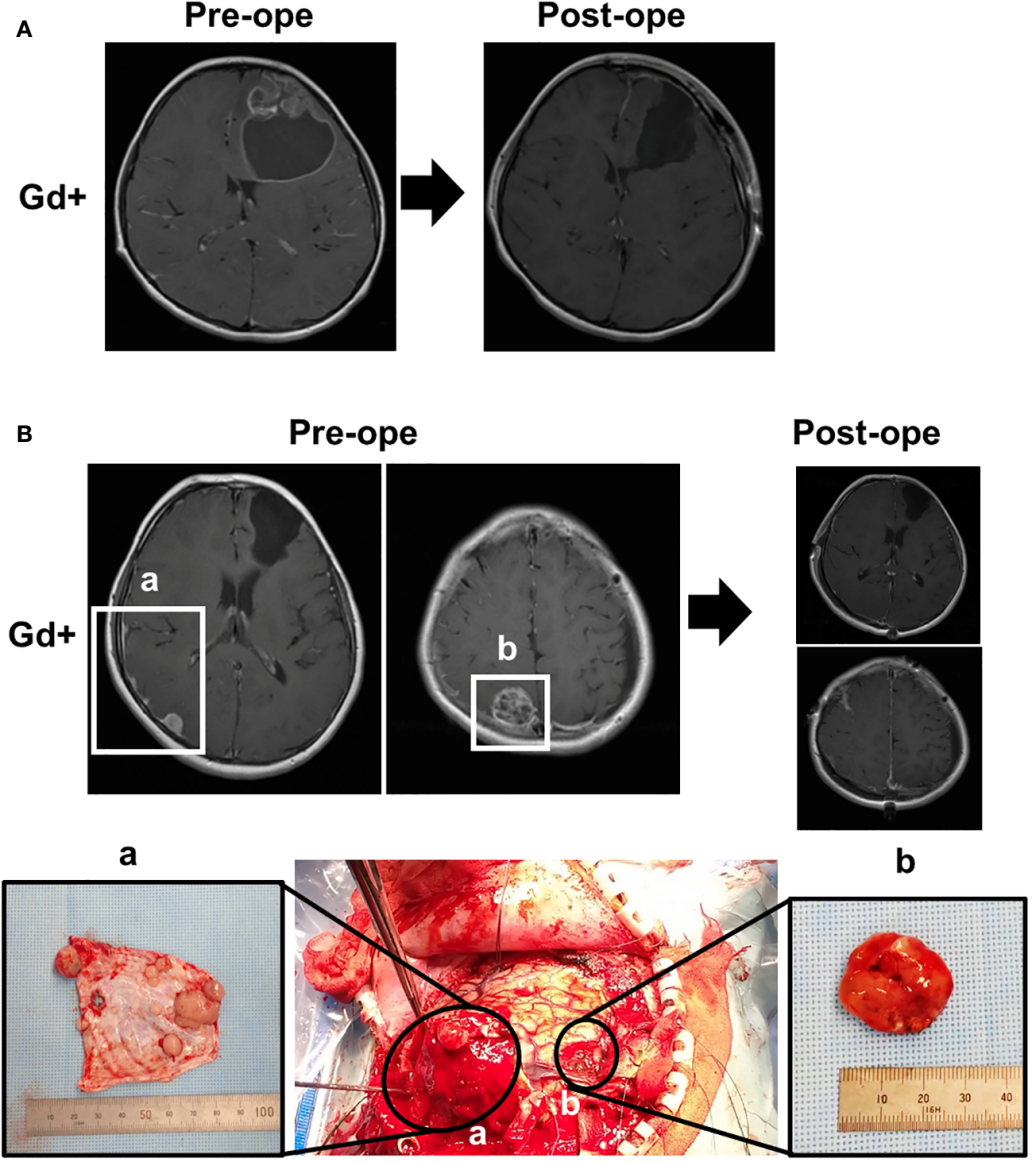
Magnetic resonance imaging (MRI) and intraoperative findings. **(A)** Preoperative and postoperative MRI at initial presentation, showing a partially contrast-enhancing lesion in the left frontal lobe with a cyst, which was completely removed by surgery. **(B)** Preoperative and postoperative MRI at the time of recurrence and correspondence with intraoperative findings. (a) Multiple small, enhanced lesions adhering to the dura, and (b) the largest heterogeneously enhanced lesion.

A 5-year-old boy with no significant medical or family history presented with headache. Magnetic resonance imaging (MRI) of the head showed a neoplastic lesion with a cyst in the left frontal lobe (**Figure 1A**), and he underwent craniotomy. Dura mater was not resected because initial tumor was mainly located within the brain parenchyma. The pathological diagnosis was anaplastic ependymoma, and the Ki-67 labeling index was about 30% at the hot spot. Immunostaining of the tumor tissue was diffusely positive for L1CAM, suggesting *ZFTA*-*RELA* fusion-positivity (6). Molecular testing was positive for *ZFTA*-*RELA* fusion, leading to an integrated diagnosis of supratentorial ependymoma, *ZFTA* fusion-positive. The patient underwent radiotherapy (59.4 Gy/33 Fr) to the resection cavity as adjuvant therapy. One year and 4 months after the initial diagnosis, a dural lesion appeared in the left temporal region, which was resected with the surrounding dura. Intraoperative findings showed that the tumor was separated from the brain parenchyma, preserving the arachnoid membrane, suggesting that invasion through the brain parenchyma was not likely. One year and 8 months after the initial diagnosis, a recurrent lesion was found in the left temporal dura and in the right ventricle, for which durotomy and endoscopic tumor resection were performed. The dural defect was replaced with polyglycolic acid felt (Durawave^®^; Gunze, Osaka, Japan). Two years after the initial onset, there was a further recurrent lesion in the left temporal region. There was no recurrence in the dura mater that was replaced with Durawave^®^; therefore, the left convexity dura, including the tumor, was extensively resected and replaced with Durawave^®^ again. Two years and 9 months after the diagnosis of the initial tumor, there was a contrast-enhanced mass in the right convexity dura. There were also small lesions in the right and left lateral ventricles and in the initial resection cavity leading to the left lateral ventricles. The patient underwent open and endoscopic tumor resection. The tumor in the left convexity dura was extensively resected and was replaced with Durawave^®^ (**Figure 1B**). Surgical findings showed that small lesions undetectable on MRI were also attached to the dura mater (**Figure 1Ba**). Tumor cells were not clearly observed in the surgical margin of the removed dura mater. The tumor in the ventricles and contiguous resection cavity was removed endoscopically, and CSF was collected at this time for cytology, which showed no evidence of tumor cells. The pathological diagnosis was recurrent anaplastic ependymoma in both the dural and intraventricular lesions, and molecular analysis showed *ZFTA*-*RELA* fusion, indicating recurrent supratentorial ependymoma, *ZFTA* fusion-positive. The patient was discharged without any complications. The body and spinal MRI showed no spinal dissemination and metastases in other organs.

### 2.1 Hematoxylin-eosin staining of the initial surgical specimen

**Figure 2:**
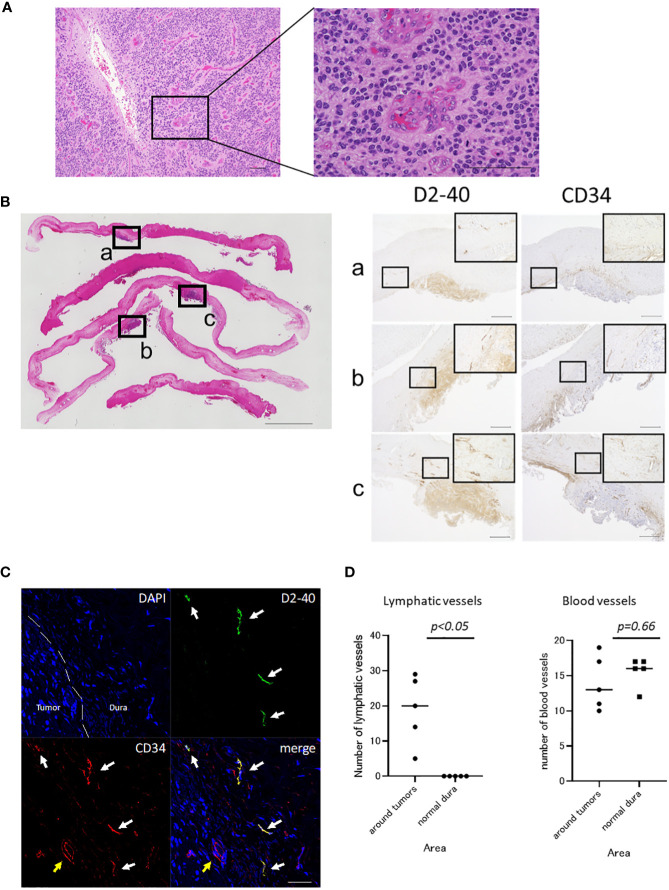
Histopathological findings of the tumor and dura. **(A)** Hematoxylin and eosin (HE) staining of the tumor at initial presentation is shown. Cells with hyperchromatic large nuclei formed a perivascular pseudorosette and proliferated (scale bar, 100 μm). **(B)** HE staining of excised dura at the time of tumor recurrence (scale bar, 5000 μm) and immunostaining for D2-40 and CD34 in serial sections (scale bar, 500 μm) are shown. The dura mater, which appeared normal macroscopically, showed tumor cells attached to the dura mater when observed microscopically after staining. In addition to D2-40^+^ tumor cells, many D2-40^+^/CD34^+^ lumens were observed in the dura at the tumor attachment site. **(C)** Fluorescence immunostaining of the dura at the tumor attachment site (scale bar, 50 μm). D2-40^+^/CD34^+^ lymphatic vessels proliferated in the dura mater of the tumor-adherent area (white arrow). Blood vessels showed D2-40^-^/CD34^+^ (yellow arrow). **(D)** The number of the lymphatic vessels at the tumor attachment site was significantly higher than that in the normal dura mater (19 vs 0, p<0.05, Mann-Whitney U test). There was no significant difference in the number of blood vessels between the tumor attachment site and normal dura mater (14 vs 15.6, p=0.66, Mann-Whitney U test). Each square (a, b, c) shown in the HE staining on the left corresponds to the respective immunostain on the right.

We observed cells with hyperchromatic large nuclei and round-like forms proliferating while forming perivascular pseudorosettes. Mitotic figures were sporadically seen, and there was prominent microvascular proliferation. The findings are consistent with anaplastic ependymoma (**Figure 2A**).

### 2.2 Hematoxylin-eosin staining of the specimen from the fifth craniotomy

Tumor cells showing similar histopathology to the specimen from the initial surgery were proliferating and attached to the dura mater. This was consistent with the recurrent ependymoma in the dura. Retraction of the fibrous tissue of the dura mater within the lesion was partially observed, at the part where it was attached to the dura mater. Specimens from lesions in the lateral ventricle also showed similar histopathology. The dura mater, which appeared normal macroscopically, also had multiple tumor lesions upon microscopic examination, showing similar histopathological features (**Figure 2B**). 

### 2.3 Immunohistochemistry

Serial sections were immunostained using anti-podoplanin antibody (1:100, D2-40; Dako, Carpinteria, CA, USA) as a marker for lymphatic endothelium and anti-CD34 antibody (ready to use, NU-4A1; NICHIREI BIOSCIENCES INC., Tokyo, Japan) as a marker of vascular endothelial cells (**Figure 2B**). In addition to luminal structures that appeared to be lymphatic vessels, the tumor was also positive for D2-40. Compared with normal dura mater, D2-40^+^/CD34 ^+^ luminal structures were increased around the dura mater in the tumor-adherent area, which were considered to be lymphatic vessels (7, 8). These vessels were not found inside the tumor. Other D2-40^-^/CD34^+^ luminal structures were observed within the tumor and dura mater and were considered to be blood vessels. Immunofluorescence staining for D2-40 and CD34 was also performed (**Figure 2C**), which showed proliferation of the D2-40^+^/CD34^+^ lymphatic vessels in the dura mater in the tumor-adherent area.

To quantify lymphatic hyperplasia in the tumor adherent dura mater, the number of lymphatic vessels was counted and compared with those in normal dura mater. Sections were viewed at 100× magnification and the areas with the highest number of D2-40 +/CD34 + lymphatic vessels (‘‘hot spots’’) were selected. Lymphatic vessels were counted in 5 high-power field (400× magnification) hot spots in each area. The dura mater at the tumor attachment site was measured in 5 areas, and 5 areas of normal dura mater were also measured and compared (**Figure 2D**). No D2-40-positive vessels were found in the normal dura mater. The number of lymphatic vessels at the tumor attachment site was significantly higher than that in the normal dura mater (19 vs 0, p<0.05, Mann-Whitney U test). There was no significant difference in the number of blood vessels between the tumor attachment site and normal dura mater (14 vs 15.6, p=0.66, Mann-Whitney U test).

### 2.4 CSF cytology collected intraoperatively

CSF cytology was normal or benign, with no evidence of dissemination.

### 2.5 Molecular analysis

**Figure 3:**
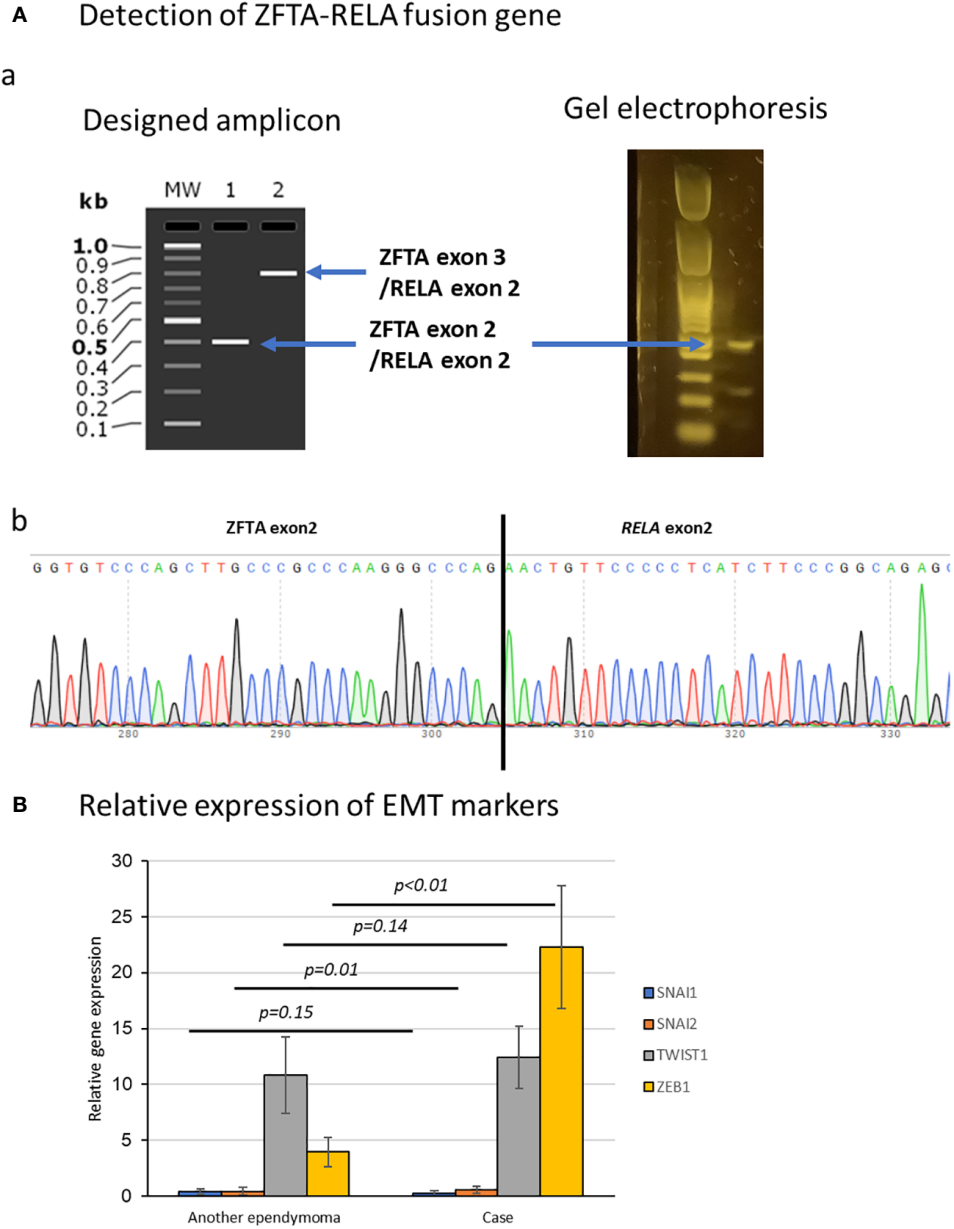
Molecular examination of the tumor. **(A)** Detection of *ZFTA-RELA* translocation in DNA extracted from specimens. (a) Electrophoresis showed a band at the position of the fusion of *ZFTA* exon 2 and *RELA* exon 2. (b) Sequence analysis also showed the *ZFTA-RELA* fusion. **(B)** Comparison of epithelial-to-mesenchymal transition factors (*SNAI1*, *SNAI2*, *ZEB1*, and *TWIST1*) in this case and another ependymoma. *ZEB1* was elevated compared with in ependymoma without dural lesions. Data represent the mean ± SEM.

IDH1-R132H: wild type, IDH2-R172H: wild type, BRAF-V600E: wild type, H3F3A-K27M, G34R/V: wild type, HIST1H3B-K27M: wild type, HIST1H3C-K27M: wild type, TERT promoter: wild type, MGMT promoter: unmethylated, ATRX-lost: retained, CDKN2A-deleted: retained, CDKN2B-deleted: retained, TP53: wild type, *ZFTA-RELA* fusion gene: detected (**Figure 3A**), ERBB1: highly expressed, ERBB2: highly expressed, TERT: highly expressed.

### 2.6 Expression of epithelial-to-mesenchymal transition factors

Expression levels of *SNAI1*, *SNAI2*, *ZEB1*, and *TWIST1*, which are factors associated with epithelial-to-mesenchymal transition (EMT) (9), were normalized to *ACTB* expression level as 100. We also examined and compared the expression levels of the same factors in another case of supratentorial ependymoma, *ZFTA* fusion-positive, CNS WHO grade 3 (without dural lesions) at our hospital (**Figure 3B**). *ZEB1* was elevated in this case compared with ependymoma without dural lesions (P<0.001).

Total RNA and genomic DNA were isolated from intact tumors using ISOGEN (NIPPON GENE, Tokyo, Japan). cDNA libraries were constructed from total RNA using ReverTra Ace^®^ qPCR RT Kit (TOYOBO, Osaka, Japan). Expression of *ACTB*, *SNAI1*, *SNAI2*, *ZEB1*, and *TWIST1* was detected by real-time quantitative polymerase chain reaction (RT-qPCR). Expression of *SNAI1*, *SNAI2*, *ZEB1*, and *TWIST1* was calculated by relative changes compared with *ACTB* using the ΔΔCt method. RT-qPCR was performed using Fast SYBR^®^ Green Master Mix kit (Applied Biosystems, USA) in ViiA™ 7 Real-Time PCR System (Applied Biosystems, Waltham, MA, USA). The amplification efficiency of each primer pair was evaluated by the standard curve method. Each sample was analyzed in triplicate. The p-values were calculated using a Student’s t-test.

The *ZFTA-RELA* fusion transcript was amplified by PCR from cDNA libraries with the following primers.

ZFTA-RELA-F: GCGCTACTACCACGACCACT

ZFTA-RELA-R: GCTGCTCAATGATCTCCACA

The length of amplicons is as follows and was confirmed by agarose gel electrophoresis.

ZFTA exon 3/RELA exon 2(791bp)

ZFTA exon 2/RELA exon 2 (404bp)

The sequence of the fusion region was confirmed by Sanger sequencing with the following primers using Sequencing Kit (BigDye™ Terminator v3.1, Applied Biosystems).

C11orf95-RELA-seq: GGCTGGAGTACCTGATGGGAC

## 3 Discussion

Lymphatic metastasis is frequent in most malignant tumors other than central nervous system(CNS) tumors; for example, lymphatic metastasis of breast cancer and malignant melanoma is well known (10, 11). Recently, it has become evident that lymphatic vessels also exist in the dura mater, and there have been a few reports on the relationship between dural lymphatics and neurodegenerative diseases (4). There have been case reports of glioma metastasizing to cervical lymph nodes, suggesting the possibility of lymphatic vessels in the dura mater, but none of them have been histopathologically proven (12, 13).

In the present case, the initial lesion was an intra-axial tumor, but recurrent lesions appeared in the dura mater on multiple occasions, presenting a recurrence pattern different from the local recurrence of the primary lesion. Surgical findings at the first recurrence showed that the arachnoid membrane was maintained between the lesion attached to the dura mater and the brain parenchyma, thus direct invasion from the tumor into the dura was unlikely. Spinal fluid cytology has remained negative so far, indicating that dissemination of the tumor was unlikely. Multiple lesions appeared in the dura contralateral to the initial lesion, thus it was suspected that the tumor had metastasized through the dura. We hypothesized that the dural lymphatic vessels, which have been the focus of much attention in recent years, might have been the route of metastasis, and performed immunostaining using lymphatic markers.

Even the dura that appeared normal macroscopically showed multiple areas of tumor cell proliferation microscopically, and each lesion was discontinuous. Therefore, we considered that the tumor cells metastasized via certain pathways, settled and proliferated at the site, rather than invading directly. In the present case, D2-40^+^/CD34^+^ lymphatic vessels proliferated in the dura at the site of tumor attachment. No tumor cells were found in these lymphatic vessels, so direct demonstration of tumor invasion into the lymphatic vessels could not be established. Another possible form of tumor metastasis could have been a CSF seeding, but this was not strongly suspected because CSF cytology was repeatedly negative. We also cannot rule out the possibility of hematogenous metastasis through the venous plexus. At least no tumor cells were found in the blood vessels of the dura either. The histopathological findings led us to suspect that the lymphatic vessels were involved in some way in the extension or growth of the tumor.

D2-40 is a monoclonal antibody against podoplanin, a transmembrane glycoprotein expressed in lymphatic epithelium (8, 14). Lymphangiogenesis is known to promote lymphatic metastasis of tumors (11, 15, 16). It has been reported that podoplanin expression is significantly correlated with lymph node metastasis in gastric cancer, and lymph vessel density may be an important prognostic factor of lymph nodes metastasis (17). Although CD34 is a marker of vascular endothelial cells (18), it is also expressed by lymphatic endothelial cells in human tumors (7). Therefore, the lymphatic vessels in our case have been tumor-associated lymphangiogenesis.

Although it is rare for CNS tumors to metastasize extracranially, there is a report of glioblastoma detected in cervical lymph nodes (12). That report does not provide direct histological evidence of tumor invasion into the dural lymphatics, but indirectly suggests that CNS tumors can also metastasize through the dural lymphatics. Deckert et al. found no lymphatic vessels in the tissue of primary central nervous system lymphoma (PCNSL) deep in the brain, while diffuse large B-cell lymphoma (DLBCL) in the dura mater showed lymphatic vessels in the dural infiltrate (19). The absence of lymphatic vessels in the lesions may explain why PCNSL and gliomas are less likely to metastasize extracranially, whereas normal DLBCL can spread easily throughout the body. Conversely, CNS tumors that once invade the dura may metastasize via lymphatic vessels. However, tumors that arise in the dura, such as meningiomas, do not often metastasize extracranially, suggesting that other factors may be involved in metastasis via the dural lymphatics.

Ependymomas arising in the dura mater are rare, with only 10 cases reported to date (20). In the present case, the primary tumor was intra-axial, but it subsequently recurred in the dura mater and ventricles, suggesting that it had something in common with the cases reported above. In the report above, two out of 10 cases were positive for the *ZFTA*-*RELA* fusion-positive, which is consistent with the present case. *ZFTA* fusion-positivity is a poor prognostic factor and may play a role in the development of dural ependymomas. EMT is a biological process that transforms epithelial cells into a mobile mesenchymal phenotype, which is known to promote cancer invasion and metastasis (9). In addition, *RELA*-fusion is reported to be associated with an EMT-like phenotype in ependymoma (21). We examined the expression of *SNAI1*, *SNAI2*, *ZEB1*, and *TWIST1*, which are known factors for EMT. The results were compared with a case of supratentorial ependymoma, *ZFTA* fusion-positive, CNS WHO grade 3 without dural involvement. *ZEB1* in this case was elevated compared to other ependymomas without dural involvement, which may have been related to tumor invasion of the dura mater.

We did not detect tumor cells in the dural lymphatics in this case. However, there was significant D2-40^+^/CD34^+^ lymphatic hyperplasia in the dura at the site of tumor attachment, indirectly suggesting that the tumor had spread via the dural lymphatics. If lymphatic metastasis had occurred, the tumor could have spread to the dura mater of the spine or to the trunk. However, we observed no such metastasis, and the recurrent lesions were confined to the convexity dura and the ventricles. The reasons why CNS tumors rarely metastasize extracranially include the presence of a blood-brain barrier, elimination by systemic immunity, and the poor prognosis of patients who die before extracranial metastasis (22, 23). In the present case, it remains to be seen whether the recurrence is confined to the intracranial region, or whether it will spread extracranially in the future. Future studies analyzing a large number of patients are warranted to confirm the findings in this case report.

## 4 Conclusion

We report a case of ependymoma with repeated recurrence in the dura mater, in which D2-40+/CD34+ lymphatic vessels proliferated in the peritumoral dura mater. As with tumors of the trunk, meningeal lymphatic vessels may be involved in metastasis of brain tumors, and further research is needed to elucidate the mechanism of this phenomenon.

## Data availability statement

The original contributions presented in the study are included in the article/supplementary material. Further inquiries can be directed to the corresponding author.

## Ethics statement

The studies involving humans were approved by Keio University School of Medicine Ethics Committee. The studies were conducted in accordance with the local legislation and institutional requirements. Written informed consent for participation in this study was provided by the participants’ legal guardians/next of kin. Written informed consent was obtained from the minor(s)’ legal guardian/next of kin for the publication of any potentially identifiable images or data included in this article.

## Author contributions

UH: Conceptualization, Data curation, Investigation, Writing – original draft. RT: Methodology, Supervision, Writing – review & editing. MY: Data curation, Investigation, Methodology, Writing – review & editing. YK: Methodology, Visualization, Writing – review & editing. NT: Visualization, Writing – review & editing. TMiy: Data curation, Writing – review & editing. JS: Data curation, Writing – review & editing. KK: Data curation, Writing – review & editing. TMiw: Supervision, Writing – review & editing. MT: Supervision, Writing – review & editing.
